# CD1C is associated with breast cancer prognosis and immune infiltrates

**DOI:** 10.1186/s12885-023-10558-2

**Published:** 2023-02-08

**Authors:** Xiao Chen, Jianzhong Zhang, Xinhan Lei, Lei Yang, Wanwan Li, Lu Zheng, Shuai Zhang, Yihan Ding, Jianing Shi, Lei Zhang, Jia Li, Tong Tang, WenJun Jia

**Affiliations:** 1 The General Surgery Department of The Second Hospital of Anhui Medical University, Hefei, China; 2 Anhui Medical University, Hefei, China

**Keywords:** CD1C, Breast cancer, Tumor microenvironment, ESTIMATE algorithm, CIBERSORT algorithm

## Abstract

**Background:**

The tumor microenvironment (TME) in breast cancer plays a vital role in occurrence, development, and therapeutic responses. However, immune and stroma constituents in the TME are major obstacles to understanding and treating breast cancer. We evaluated the significance of TME-related genes in breast cancer.

**Methods:**

Invasive breast cancer (BRCA) samples were retrieved from the TCGA and GEO databases. Stroma and immune scores of samples as well as the proportion of tumor infiltrating immune cells (TICs) were calculated using the ESTIMATE and CIBERSORT algorithms. TME-related differentially expressed genes (DEGs) were analyzed by a protein interaction (PPI) network and univariate Cox regression to determine CD1C as a hub gene. Subsequently, the prognostic value of CD1C, its response to immunotherapy, and its mechanism in the TME were further studied.

**Results:**

In BRCA, DEGs were determined to identify CD1C as a hub gene. The expression level of CD1C in BRCA patients was verified based on the TCGA database, polymerase chain reaction (PCR) results, and western blot analysis. Immunohistochemical staining (IHC) results revealed a correlation between prognosis, clinical features, and CD1C expression in BRCA. Enrichment analysis of GSEA and GSVA showed that CD1C participates in immune-associated signaling pathways. CIBERSORT showed that CD1C levels were associated with tumor immune infiltrating cells (TILs), such as different kinds of T cells. Gene co-expression analysis showed that CD1C and the majority of immune-associated genes were co-expressed in BRCA. In renal cell carcinoma, patients with a high expression of CD1C had a better immunotherapy effect.

**Conclusion:**

CD1C is an important part of the TME and participates in immune activity regulation in breast tumors. CD1C is expected to become a prognostic marker and a new treatment target for breast cancer.

**Supplementary Information:**

The online version contains supplementary material available at 10.1186/s12885-023-10558-2.

## Introduction

Globally, breast cancer is the most prevalent malignant tumor in women. The International Cancer Research Center showed that in the female population, approximately 230 new cases of breast cancer were diagnosed in 2020, which surpassed the incidence rate of lung cancer world [1]. Invasive breast cancer (BRCA) is a common histological type of breast cancer. In BRCA, cancer cells generally penetrate the basement membrane of breast ducts or lobular acini and infiltrate into the stroma, with a high degree of malignancy [2]. Currently, immunotherapy is a new therapeutic direction for breast cancer, but the key therapeutic target remains to be found.

The tumor microenvironment (TME) has recently emerged as a vital player in the progression of breast cancer, with the potential as a future treatment target [3]. Apart from cancer cells, the TME is made up of immune cells, cells composing blood vessels, stromal cells, and the extracellular matrix (ECM) [4]. Stromal and immune cells are involved in tumor progression [5].

This study collected gene expression data of breast tumor samples from the TCGA and GEO databases. The ESTIMATE algorithm was used to determine immune, stromal, as well as ESTIMATE scores and provide an overall view of the TME [6]. A high TME immune score correlates with a better survival in breast cancer [7]. In addition, we explored differentially expressed genes (DEGs) based on stromal and immune score groups. Gene intersection was screened by overlapping between DEGs and the genes obtained by Cox regression analysis through a protein interaction (PPI) network to determine CD1C as a hub gene.

CD1C is located on the surface of dendritic cells (DCs). The gene belongs to the CD1 family, which is structurally related to major histocompatibility complex (MHC) proteins [8]. CD1C proteins mediate the presentation of Class I MHC antigen to T cells [9]. CD1C plays vital roles in immune diseases and contributes to the etiology of these diseases [7, 10]. CD1C levels are elevated in gastric cancer patients [11]. In non-small cell lung cancer (NSCLS), CD1C plays an antitumor role in vivo [12]. Through the TCGA database, CD1C was verified to have low expression in BRCA. Elevated CD1C levels correlated with good prognostic outcomes for breast cancer patients, implying that CD1C is involved in inhibition of tumor progression. Further enrichment analysis of GSEA and GSVA showed that CD1C plays a role in a variety of immune associated pathways. The CIBERSORT algorithm was used to calculate tumor immune infiltrating cell (TIL) subsets in the TME of BRCA [13]. It was revealed that CD1C was associated with a variety of TILs and co-expressed with immune-related genes. These findings suggested that CD1C plays a vital role in regulating the TME and is a potential new prognostic marker and promising treatment target for BRCA.

## Materials and methods

### Ethical approval

The Institutional Research Ethics Committee of The Second Affiliated Hospital of Anhui Medical University approved this study. All patients signed written informed consent. The ethical approval number is No. AHMU2ND-2021-23. The present study evaluated 105 patients subjected to radical mastectomy at The Second Affiliated Hospital of Anhui Medical University (Hefei, China) between January 2017 and December 2022, with a postoperative pathological diagnosis of BRCA. Patients that had been subjected to radiotherapy or chemotherapy prior to surgery were exempted from the analyses.

### Data preparation

**Fig. 1:**
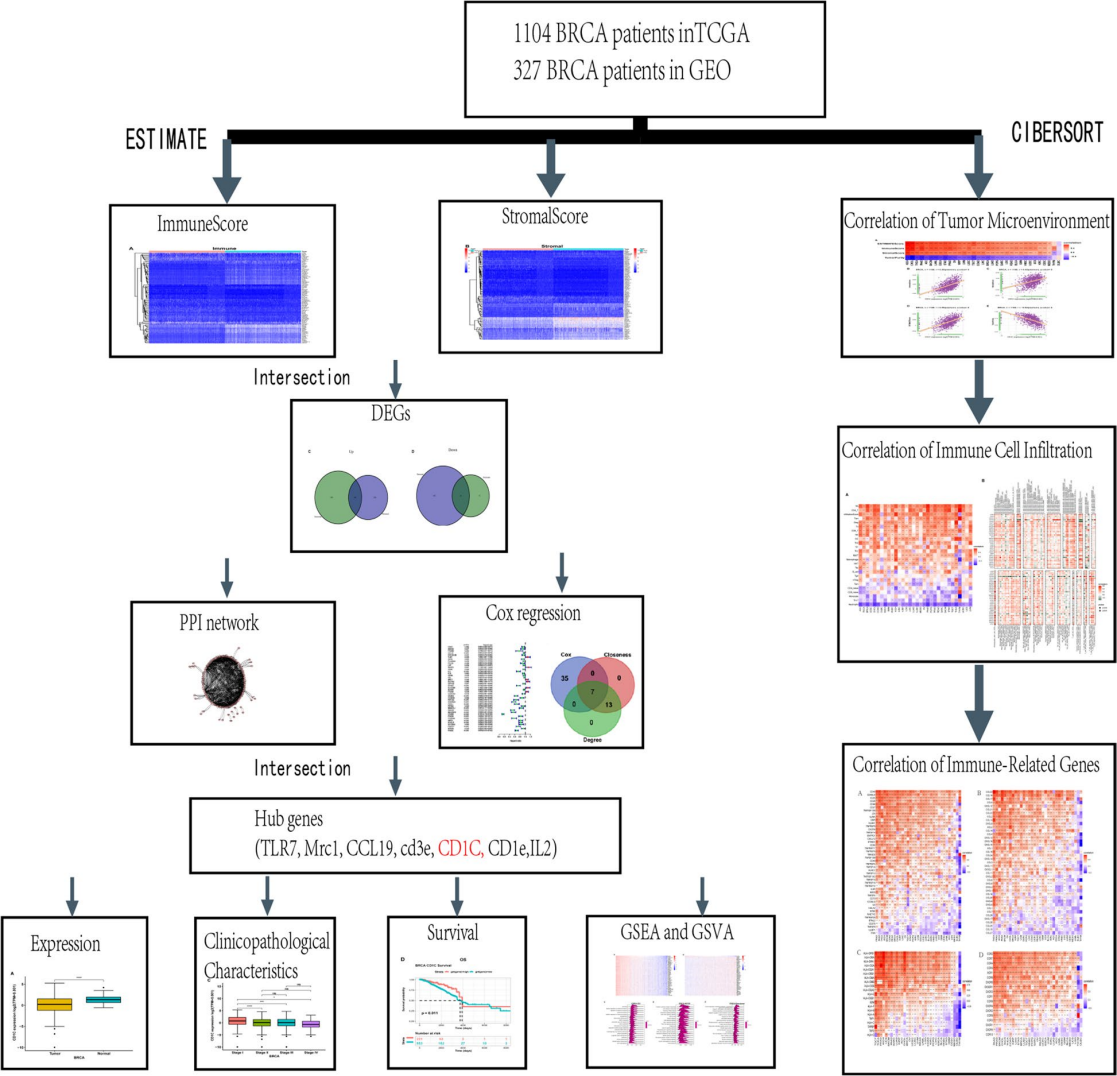
Flowchart of this study

The specific research steps are shown in the flowchart in Fig. 1. The RNA sequencing data and clinical data of 1104 breast cancer patient samples and 327 samples from Gene Expression Omnibus were retrieved for analysis. Transcriptional values were log2-transformed using the “limma” package in the R language. The clinical information of the corresponding patients was used for subsequent analysis, such as survival time and status, age, and pathological characteristics.

### Clinic-pathological characteristic and survival analyses

The infiltration level of stromal cells and immune cells in the breast cancer TME was evaluated by the ESTIMATE algorithm. The data from TCGA and GSE20698 were merged and batch effects eliminated using the “SVA” package in R [14]. “Estimate” in R [6] was used to carry out immunization and stroma scores for all breast cancer patients. All samples were assigned into two groups based on stromal, immune, and ESTIMATE scores. The intermediate value of the score was considered as the boundary value for dividing different groups. We compared the clinicopathological features of different groups, including age, stage, and TNM classification. Furthermore, the association between the immunity, stroma, and ESTIMATE scores as well as survival rates of BRCA patients were analyzed through the Kaplan-Meier method.

### Identification analysis

In this study, samples were assigned to high- and low- stromal and immune score groups. By analyzing the scores of different groups and intersecting them, overlapping genes based on ESTIMATE findings was selected. “Limma” in R was used for gene expression screening and to identify DEGs. “Pheatmap” in R was used to draw heatmaps of DEGs [15]. “Venn diagrams” in R was used to determine overlapping DEGs [16].

### Gene enrichment analysis

Gene enrichment analysis was used to determine potential gene functions. The packages "ggplot2" [17], "enrich plot", and "cluster profiler" in the R language were used for DEG enrichment analysis.

### PPI network and cox regression analysis

The String database was used to further analyze interactions between DEGs. The Cytoscape software was used for network visualization. We further used two algorithms: the Closeness algorithm and the Degree algorithm. Cox univariate regression analysis was performed on all genes to determine prognostic DEGs. The "Survival" package was used to indicate intersections of genes screened by Cox regression analysis and genes screened by PPI. Through screening of gene intersections, CD1C was determined as a hub gene.

### Analysis of the relationships between CD1C, prognosis, and clinical characteristics in BRCA

CD1C expression, clinical feature information, and survival data were extracted from breast cancer samples downloaded from TCGA. First, we verified the mRNA expression of differential CD1C in BRCA. Using the database, the association between CD1C levels and patient prognosis can be studied in many published cancer data sets. The analysis of the relationship between CD1C and clinical features, including age, TNM classification, and tumor stage, was conducted through the packages "limma" and "ggpubr" in R.

### Enrichment analysis of CD1C expression in BRCA

Gene Set Enrichment Analysis (GSEA) [18] and Gene Set Variation Analysis (GSVA) [19] were conducted to analyze the biological functions of CD1C.

With regards to correlation analyses of all genes in breast cancer, we used the R package “pheatmap” to show the positive and negative top-50 gene expression thermograms. Furthermore, based on the results of correlation analysis, “clusterpofiler” in R was used for GSEA enrichment analyses, and the top-20 results were displayed.

GSVA scores were generated for all tumors. “GSVA” in R was used to evaluate the relationship between CD1C in pan-cancer and 50-star pathways in hallmark gene sets. The R package “ggplot2” was used for mapping the most significant positive and negative correlations.

### Evaluation of correlation between the CD1C gene and immune infiltrations

First, to evaluate the association between CD1C and the TME, we determined the correlation between the gene and immune and stromal scores, and the tumor purity in pan-cancer as well as breast cancer. Next, to assess the relationship between the genes and immune cell infiltrations, "CIBERSORT” was used to calculate relative scores for immune cells in pan cancers using the ImmueCellAI database. Pan-cancer immune infiltration data from the TIMER2 database were used to verify the correlation. In addition, we conducted co-expression analyses of CD1C and immune-associated genes, including the immune activating gene, chemokine, chemokine receptor, and MHC gene using “limma” in R. “reshape2” and “RColorBreyer” were used to visualize the results. We further analyzed the prognosis of some immune-related genes in BRCA. Moreover, we used the TIDE database to evaluate the effect of CD1C on the prognosis of immunotherapy patients.

### Real-time quantitative reverse transcription polymerase chain reaction (qRT-PCR)

To verify the gene expression of CD1C, samples from BRCA patients were detected by qRT-PCR. Total RNA in tumor tissues and adjacent tissues was extracted by TRIzol reagent (Invitrogen, Germany). Oligo dT primers and reverse transcriptase were used to generate first strand cDNA (Invitrogen, USA) from total RNA. QuantiTect SYBR Green PCR Master Mix (Qiagen, Germany) and specific primers were used in ABI Prism 7000 analyzer (Applied Biosystems, USA) for quantitative real-time PCR (qRT-PCR). The primers were designed and synthesized by Qingke (Shanghai, China). The primer sequence is shown in Supplementary Table 1. The 2-ΔΔ Ct value was used to reflect the expression level of CD1C.

### Western blotting

Western blot analysis was used to evaluate the differential expression of the CD1C protein in tumor and adjacent tissues. Tissue samples obtained from BRCA patients were immediately frozen in liquid nitrogen and stored at - 80 °C. The sample were centrifuged at 4 °C and 12,000 rpm for 30 min. The supernatant was collected and the protein quality quantified by the BCA method. The protein lysate was boiled in SDS sample buffer at 95 °C for 10 min, electrophoresis was performed on 12% SDS PAGE gel, and the eluate was transferred to a polydifluoroethylene membrane (Millipore, USA). The membrane was sealed in 5% (w/v) skimmed milk solution at room temperature for 2 h and incubated with primary antibody (anti-CD1C antibody, ab5, Abcam, UK) at 4 °C overnight. After three times of washing in TBST buffer solution for 30 min, the membrane was incubated with secondary antibody (horseradish peroxidase bound anti rabbit IgG, dilution 1:1000; Beyotime Biotechnology, China) at room temperature for 1 h. It was then three times washed with TBST buffer solution for 30 min. The membrane was detected by an enhanced chemiluminescence method using Lumi Glo reagent (Millipore, USA). Image J software was used to quantify immunoblotting.

### IHC staining

Faure Marin fixed, paraffin embedded breast tumor tissues and matched normal tissues were obtained from primary breast cancer patients and used for immunohistochemical staining. The main antibodies used were as follows: anti-CD1C (1:200; Abcam), CD4 (1:200; Abcam), CD8 (1:200; Abcam), CCL19 (1:200; Abcam), and Ki-67 (1:300; Abcam).

The tissues were sectioned into 3-µm thick slices that were heated in a 57-℃ incubator for 90-120 min, dewaxed using xylene, diluted in a series of concentrations of ethanol, and rehydrated. They were then boiled in citrate buffer (0.01 M, pH 6.0) for 2 min. Endogenous hydrogen peroxide activities were blocked using 0.3% hydrogen peroxide. Primary antibody was incubated overnight at 4 °C. After removal, it was washed thrice (5 min each) in 0.01 mol / L PBS, and then cultured with biotin-labeled secondary antibody at room temperature (RT) for 1 h. Color development was achieved by 3,3-diaminobenzidine tetrahydrochloride (DAB), while hematoxylin was used for deposition in the nucleus.

IHC results were evaluated by combining intensity scores of staining intensity and area. A positive reaction was a reaction that showed a brown signal. The IHC score was determined by multiplying the staining intensity score (0, negative; 1, weak; 2, medium; 3, strong) by the score or positive area (0, less than 5%; 1, 5 to 25%; 2, 26 to 50%; 3, 51 to 75%; 4, more than 75%). A score of 0 to 7 indicated low expression while a score of 8 to 12 indicated high expression.

### Statistical analysis

Data processing and analysis was performed using the R (version 4.0.2) software. Perl (version 5.32.0) was used to extract data from the datasets. The statistical methods, public databases, and R software packages used are described in the methods section. *P* < 0.05 denoted significance.

## Results

### Correlation of breast, Immune, and ESTIMATE scores with survival and clinicopathological features

**Fig. 2:**
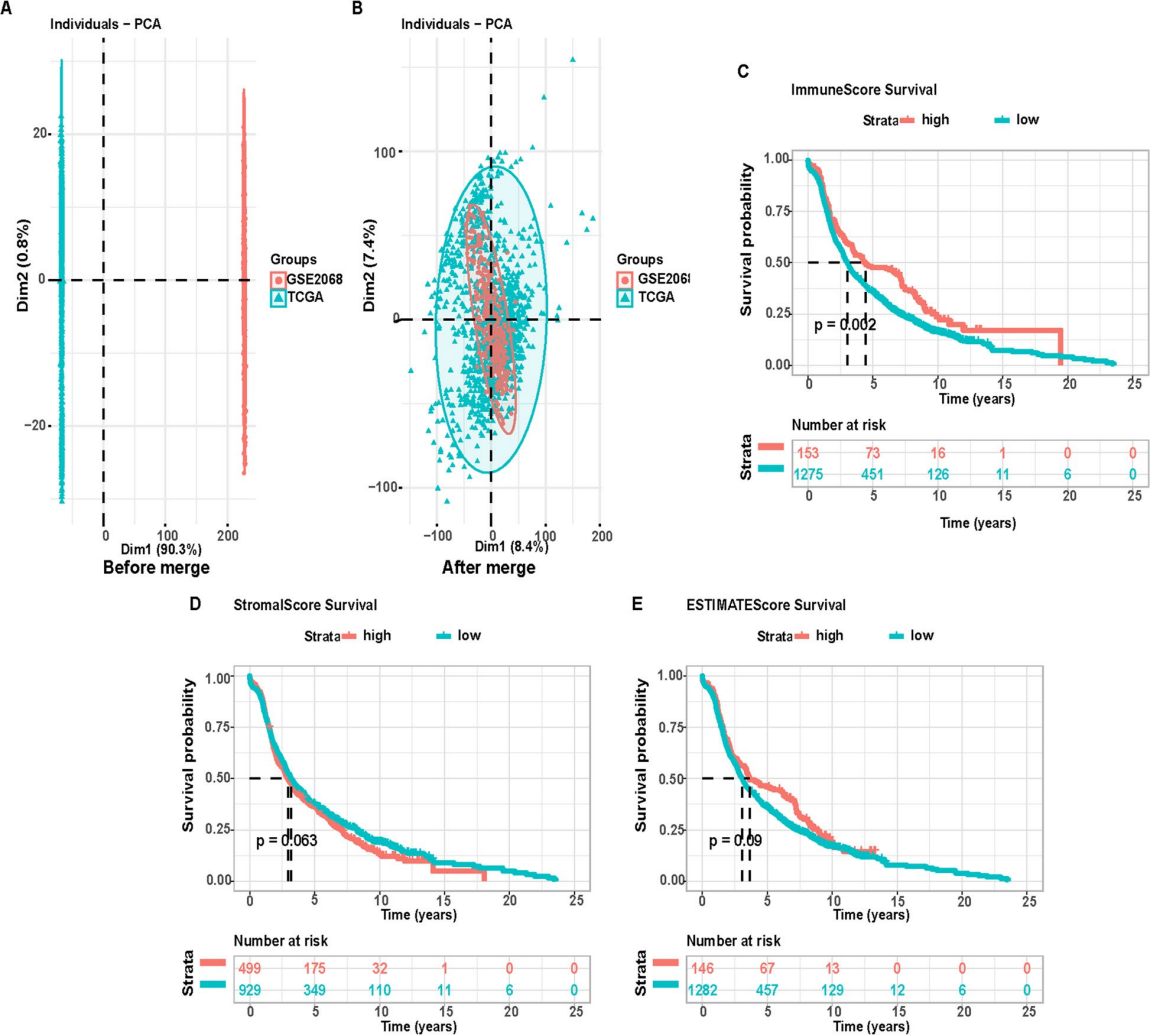
**A** PCA diagram (before consolidation), **B** PCR diagram (combined), **C**–**E** correlation of stromal, immune, and ESTIMATE scores with the survival of BRCA patients

**Fig. 3:**
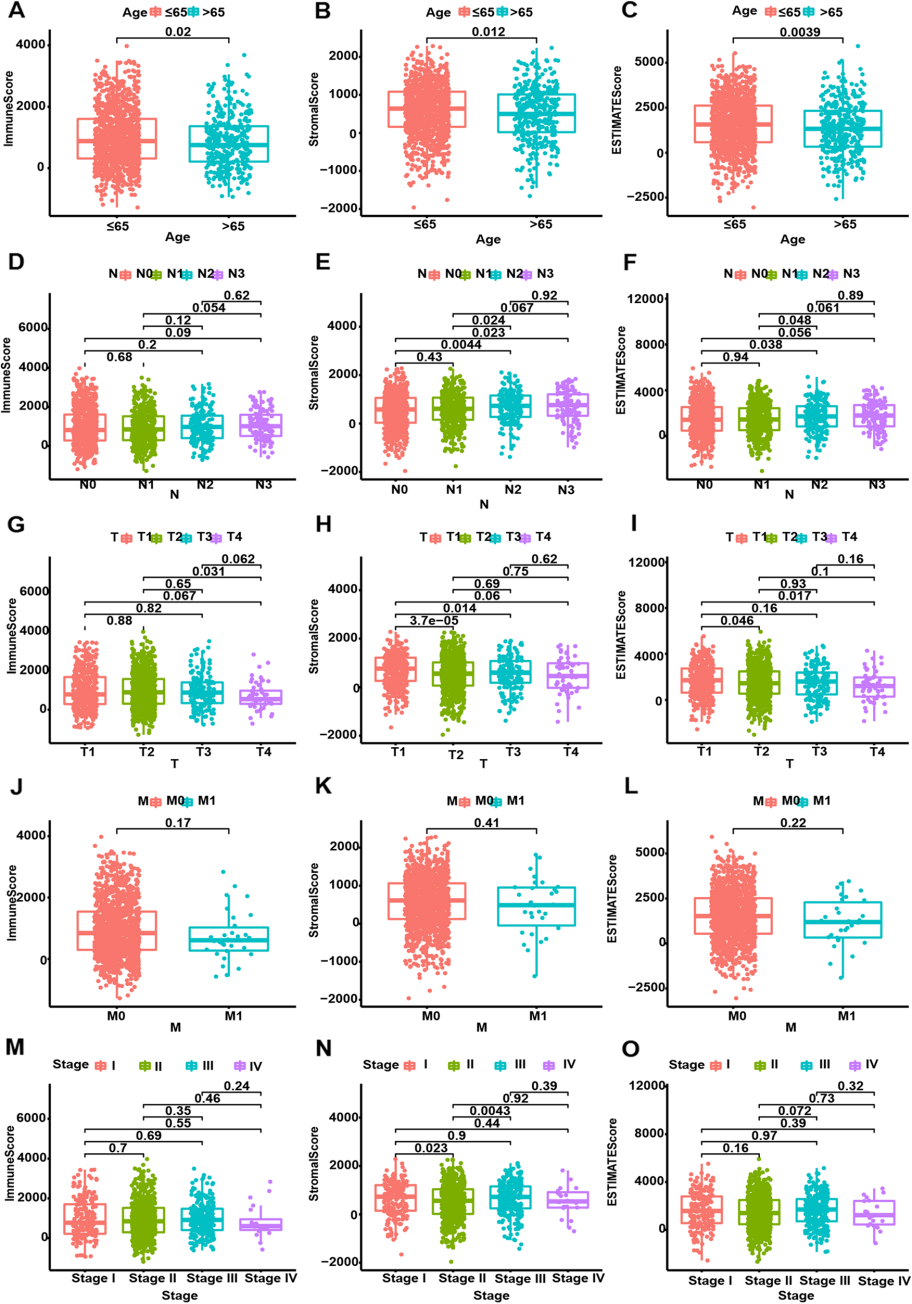
Relationship between stromal, immune, and ESTIMATE scores and clinical characteristics of BRCA patients (**A**–**O**).

We calculated the immune, stromal, and ESTIMATE scores of BRCA samples from the TCGA and GEO databases by the ESTIMATE algorithm (Fig. 2A). Then, the sample data from the two databases were merged and homogenized (Fig. 2B). The samples were divided into two groups for survival analysis (Fig. 2C-E). Survival curves showed that relative to the low-score group, the immune high-score group had a higher survival rate (Fig. 2C). Thus, levels of immune cells in BRCA may be a factor related to prognostic outcomes. Furthermore, the clinical characteristics of different groups were compared, showing that the above scores were significantly correlated with age and N and T classifications (Fig. 3).

### DEG identification and enrichment analysis

**Fig. 4:**
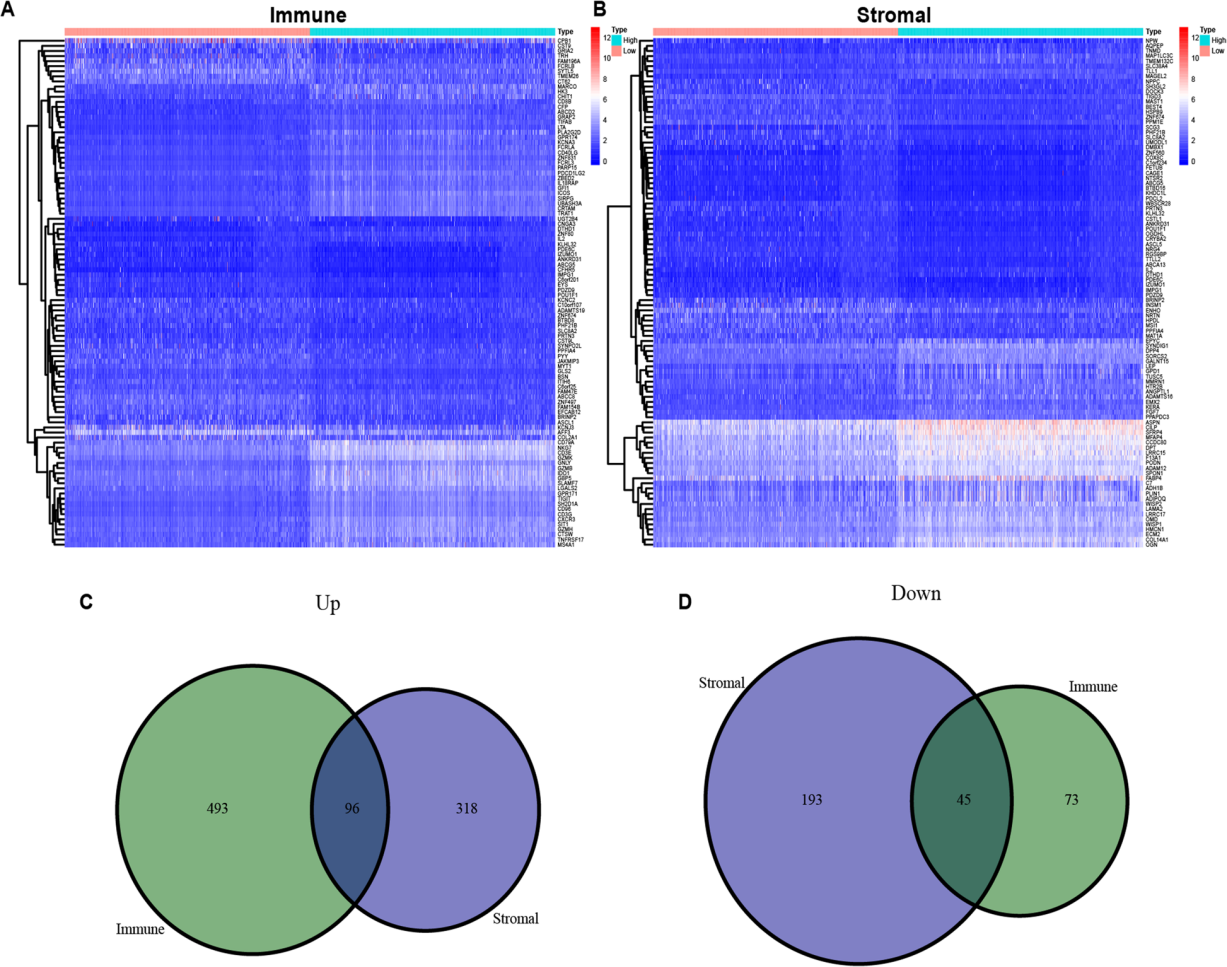
DEGs were determined by stromal and immune scores in BRCA. **A** Heatmap of genes in the immune score groups. **B** Heatmap of genes in the stromal score groups. **C** Venn diagram of the elevated stromal as well as immune score groups. **D** Venn diagram of the suppressed stromal and immune score groups

To assess the significance of the TME in BRCA, the gene expression of high and low samples was analyzed to determine DEGs (Fig. 4A and B). After systematic data standardization, 96 elevated and 45 suppressed genes were identified from the intersection of two groups. Finally, 141 DEGs in both stromal as well as immune score groups were noted as TME-associated DEGs (Fig. 4C and D).

**Fig. 5:**
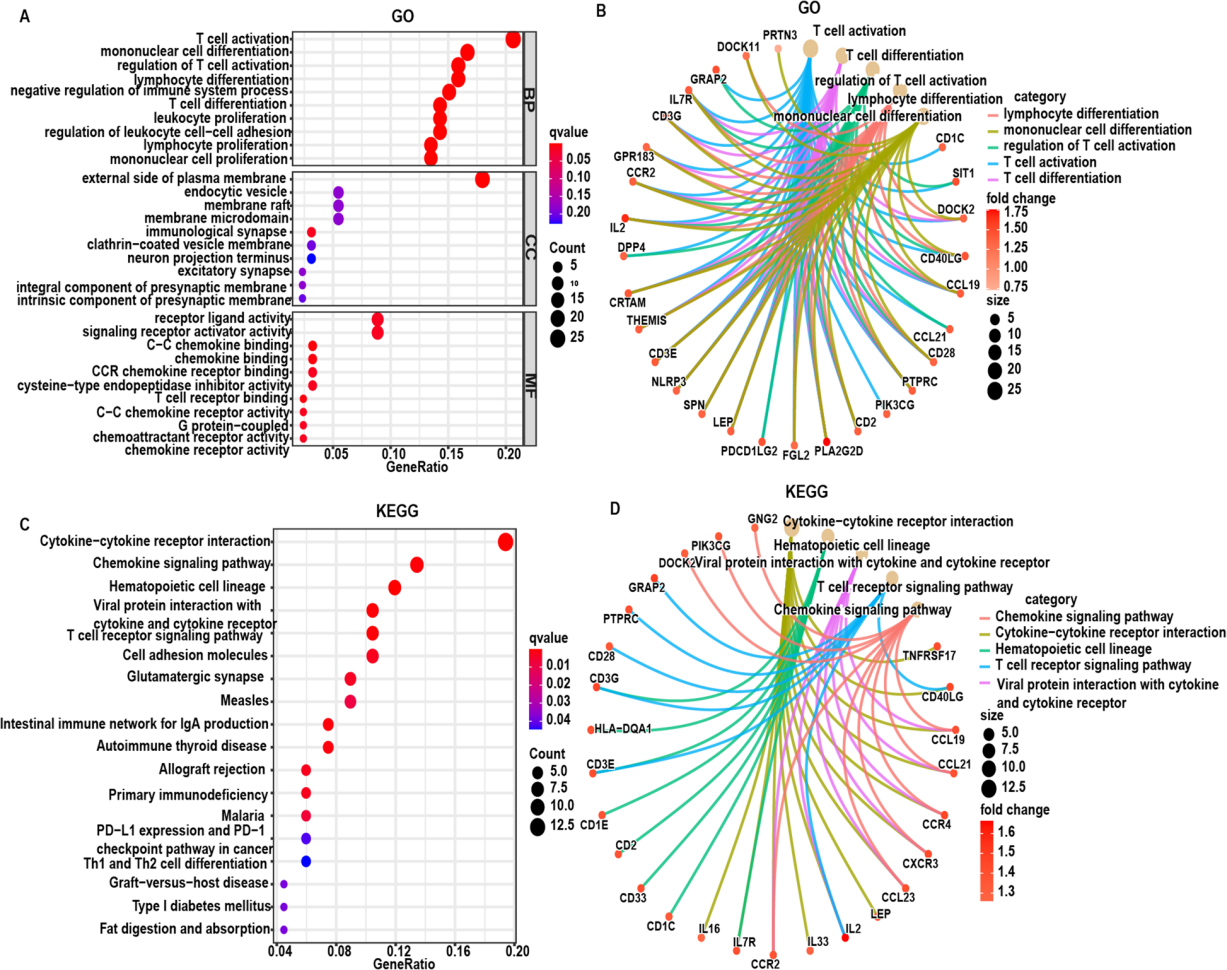
Enrichment analysis of DEGs. **A** Bubble of GO enrichment analysis; **B** Circos of top-5 Go enrichment results and related genes; **C** Bubble of KEGG enrichment analysis; **D** Circos of top-5 KEGG enrichment results and related genes

To understand the biological functions of the above DEGs, online biological databases were used for enrichment analysis. Gene Ontology (GO) analysis showed that the 141 DEGs corresponded to immune-associated GO terms, such as activation of T cells, mononuclear differentiation, and T cell activation regulation (Fig. 5A and B). Kyoto Encyclopedia of Genes and Genomes (KEGG) analysis also showed that DEGs were enriched in immune-associated pathways, including cytokine-cytokine receptor interactions and the chemokine signaling pathway (Fig. 5C and D). Thus, immune factors in the TME are involved in progression of BRCA.

### PPI network and Cox regression analysis

**Fig. 6:**
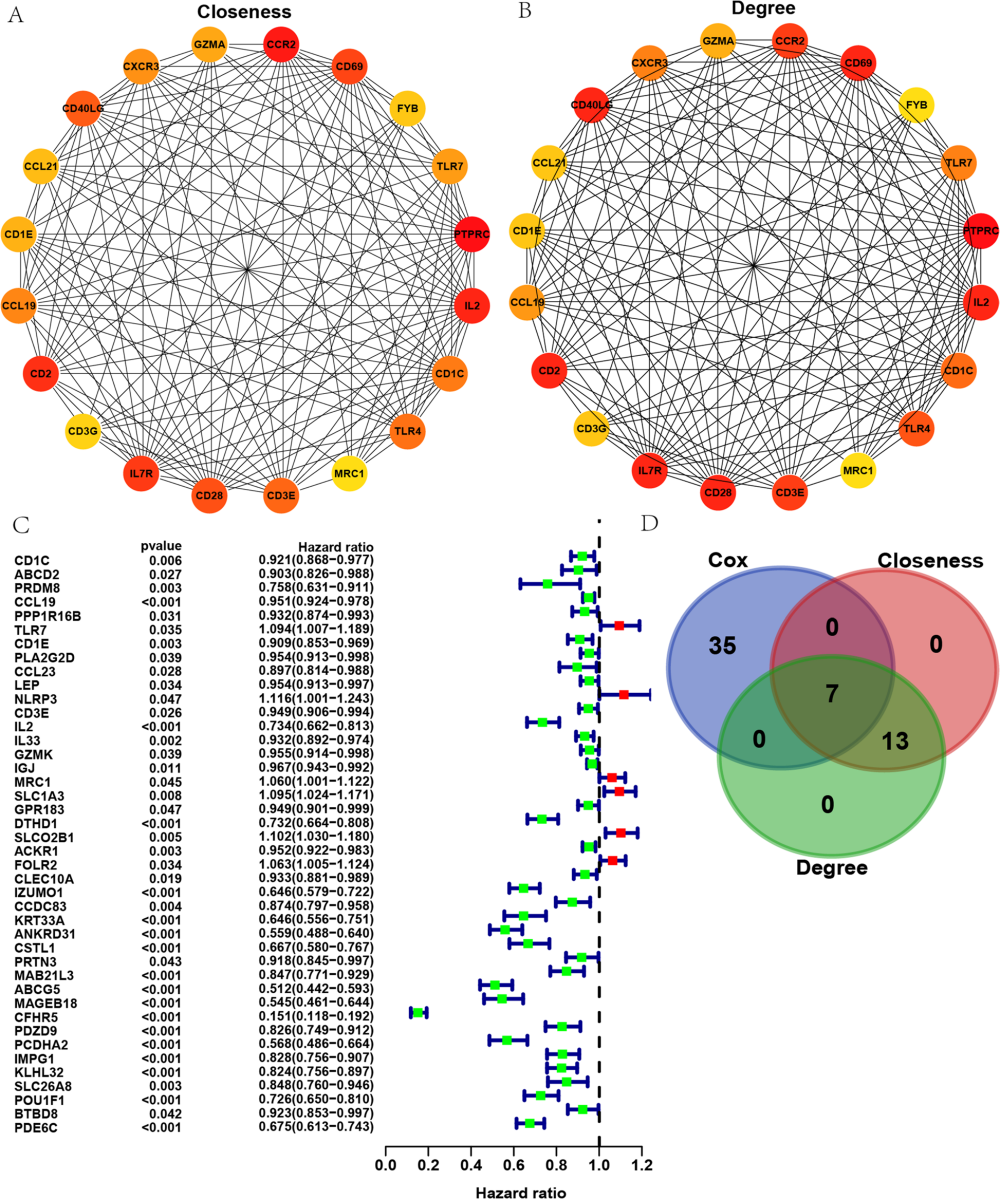
Cross screening genes through PPI network and Cox regression analysis. **A** Top-20 genes from the Degree algorithm; **B** Top-20 genes from the Degree algorithm; **C** Forest plot of univariate Cox regression analysis; **D** Venn diagram of the gene intersection between the PPI network and Cox prognostics

The STRING database was used to establish the PPI network, and DEGs were screened out. The Cytoscape software was used for visualization (Supplementary Fig. 1). From the 96 elevated and 45 suppressed genes, genes related to clinical outcomes were searched. Using the Closeness and Degree algorithms, the top-20 genes were identified by calculating the connection degree of each node in the PPI network (Fig. 6A-B). Next, 42 prognostic DEGs were determined through univariate Cox regression analysis (Fig. 6C). The top 42 factors ranked by the univariate Cox regression *P*-value, the top-20 hub genes from the Closeness algorithm, and the top-20 hub genes from the Degree algorithm were cross-analyzed to obtain TLR7, MRC1, CCL19, CD3E, CD1C, CD1E, and IL2 (Fig. 6D).

Survival analysis of the above seven genes was carried out and curves were drawn. We found that these genes were all significantly related to the survival of BRCA patients (Supplementary Fig. 2). Furthermore, we assessed the relationship between genes and clinical features (Supplementary Fig. 3). Using TCGA data to detect seven genes, it was indicated that CD1C was differentially expressed and CD1C levels were significantly associated with clinical characteristics and prognosis of BRCA patients at the same time. Therefore, we concluded that CD1C was a hub gene.

### Correlations of survival and clinic-pathological features with CD1C levels

**Fig. 7:**
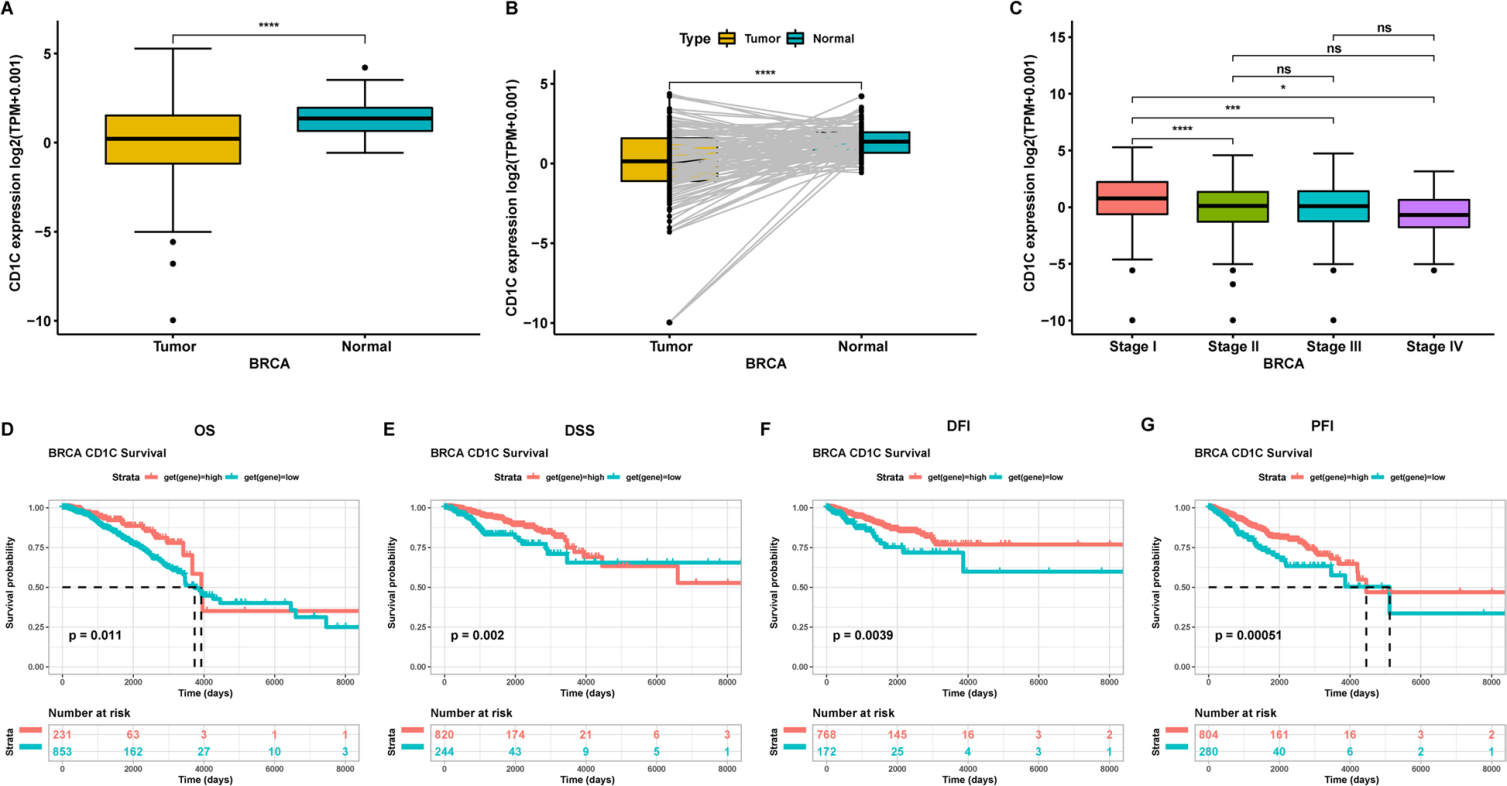
**A** Differential expression of breast cancer data from TCGA; **B** Paired expression of breast cancer data from TCGA; **C** Staging of breast cancer data from TCGA; **D**-**G** Breast cancer data survival analysis (using the best cutoff)

By analyzing CD1C gene expression of BRCA samples from TCGA, we found that CD1C levels in tumor tissues were markedly lower than in normal tissues (Fig. 7A). In further paired analysis, CD1C levels in tumor samples were markedly lower than in normal samples (Fig. 7B).

The association between CD1C levels and tumor stage was assessed. Interestingly, significant differences in CD1C expression occurred between stage I tumors and tumors of other stages (Fig. 7C). The association between CD1C levels and other clinic-pathological features showed that the expressions of CD1C was related to age, and T and M classifications (Supplementary Fig. 3B). The general trend is that the expression of CD1C is higher in early breast cancer. We speculate that this is due to the fact that the tumor is full of cancer cells and lacks immune cells and immune factors in late breast cancer.

All samples were assigned to two groups based on CD1C levels. A Kaplan-Meier survival curve showed that patients with elevated CD1C levels had a longer disease-free interval (DFI) and a longer progression free interval (PFI) relative to those with suppressed CD1C levels (Fig. 7D-G). Analysis of the data revealed that CD1C may play a protective role and lead to a good prognosis in BRCA.

### GSEA and GSVA

**Fig. 8:**
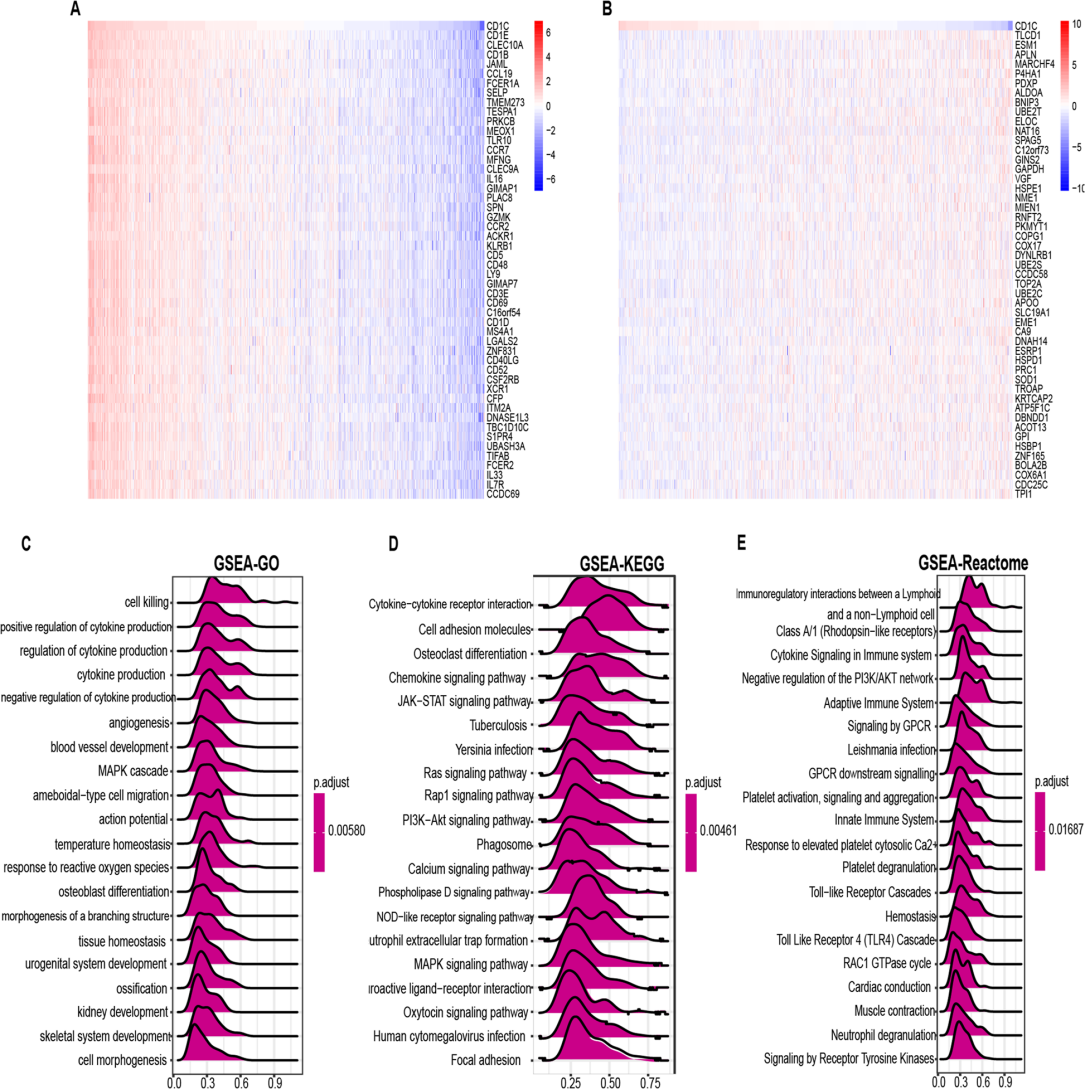
GSEA enrichment analysis. **A** Positive correlation top-50 gene expression heat map; **B** Heat map of negative correlation top-50 gene expression; **C**-**E** GSEA enrichment analysis and top-20 results

**Fig. 9:**
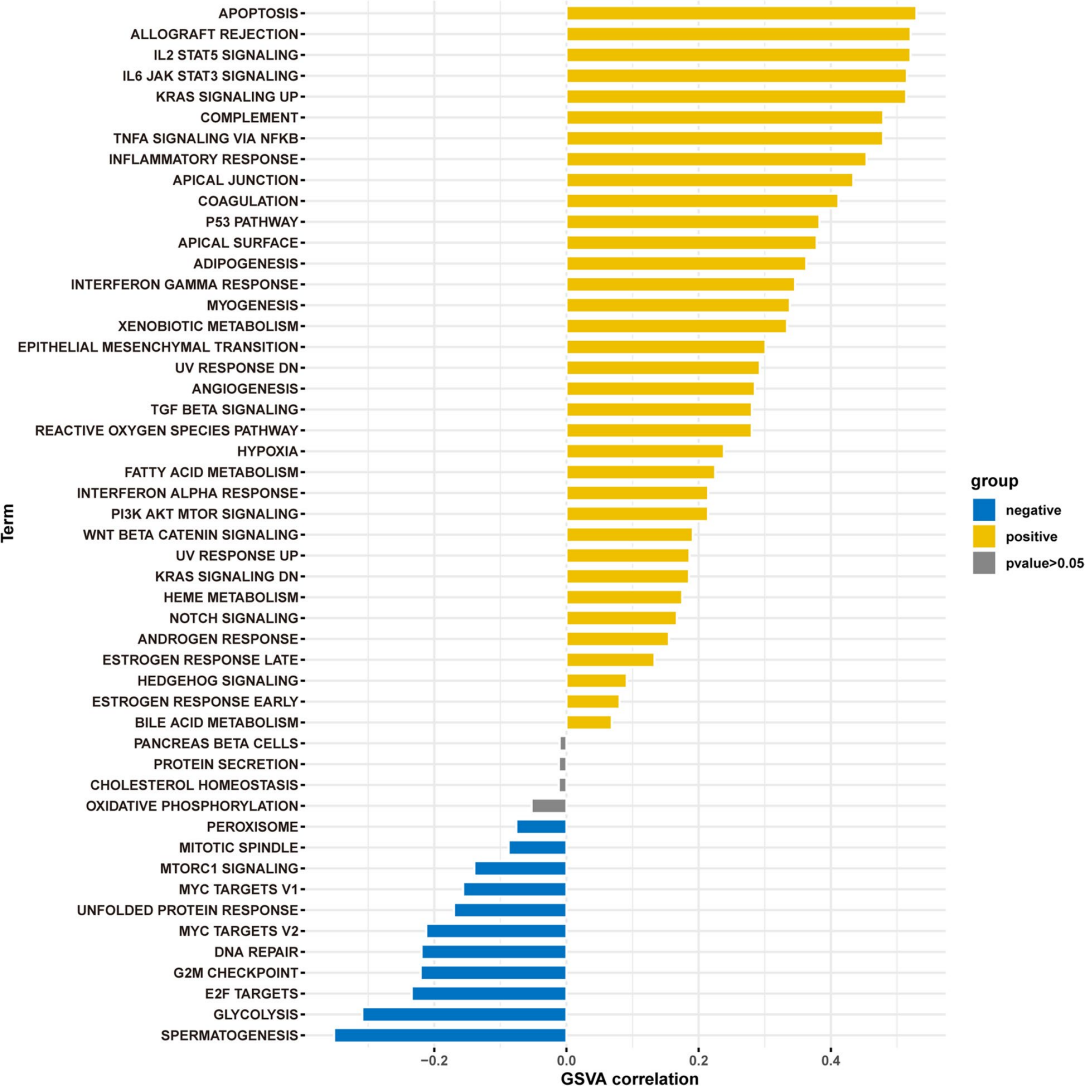
GSVA enrichment analysis of correlation between CD1C in pan cancer and 50-star pathways in hallmark

CD1C was revealed to play a role in immune-associated signaling pathways, such as regulation of cytokine production, cytokine-cytokine receptor interactions, and immunoregulatory interactions between non-lymphoid and lymphoid cells (Fig. 8). The correlation of CD1C expression with 50-star pathways in each tumor was analyzed using GSVA. The results demonstrated that CD1C was positively associated with several pathways, including apoptosis, allograft rejection, IL-2 Signaling, and IL-6 Signaling (Fig. 9).

### Relationship between CD1C expression and the TME

**Fig. 10:**
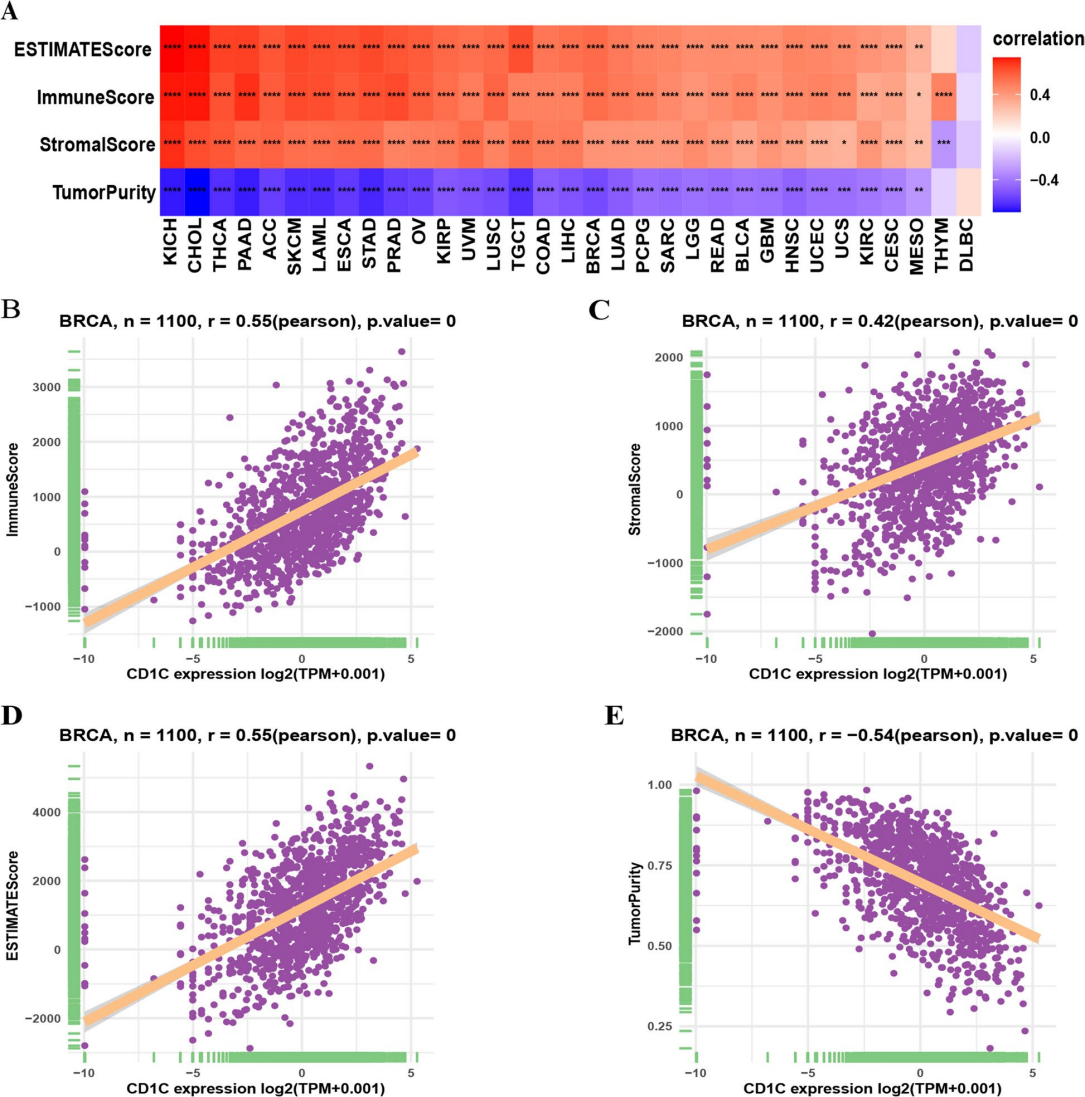
Correlation between CD1C and the TME. **A** The correlation between CD1C and ESTIMATE, immune as well as stromal scores and tumor purity in pan cancer; **B**-**E** Association between CD1C and ESTIMATE, immune, and stromal scores and tumor purity in breast cancer

To explore the correlation between CD1C and the TME, we analyzed the relationship between the CD1C gene and immune, stromal, and ESTIMAE scores and tumor purity in pan cancer. The levels of CD1C were positively correlated with immune, stromal, as well as ESTIMATE scores, and negatively correlated with tumor purity (Fig. 10A). Further analysis showed that the correlation between CD1C and the above scores in BRCA was also statistically significant (Fig. 10B-E). This suggested that CD1C levels are closely related to the immune-activated status of BRCA.

### Association between CD1C levels and immune cell infiltration

**Fig. 11:**
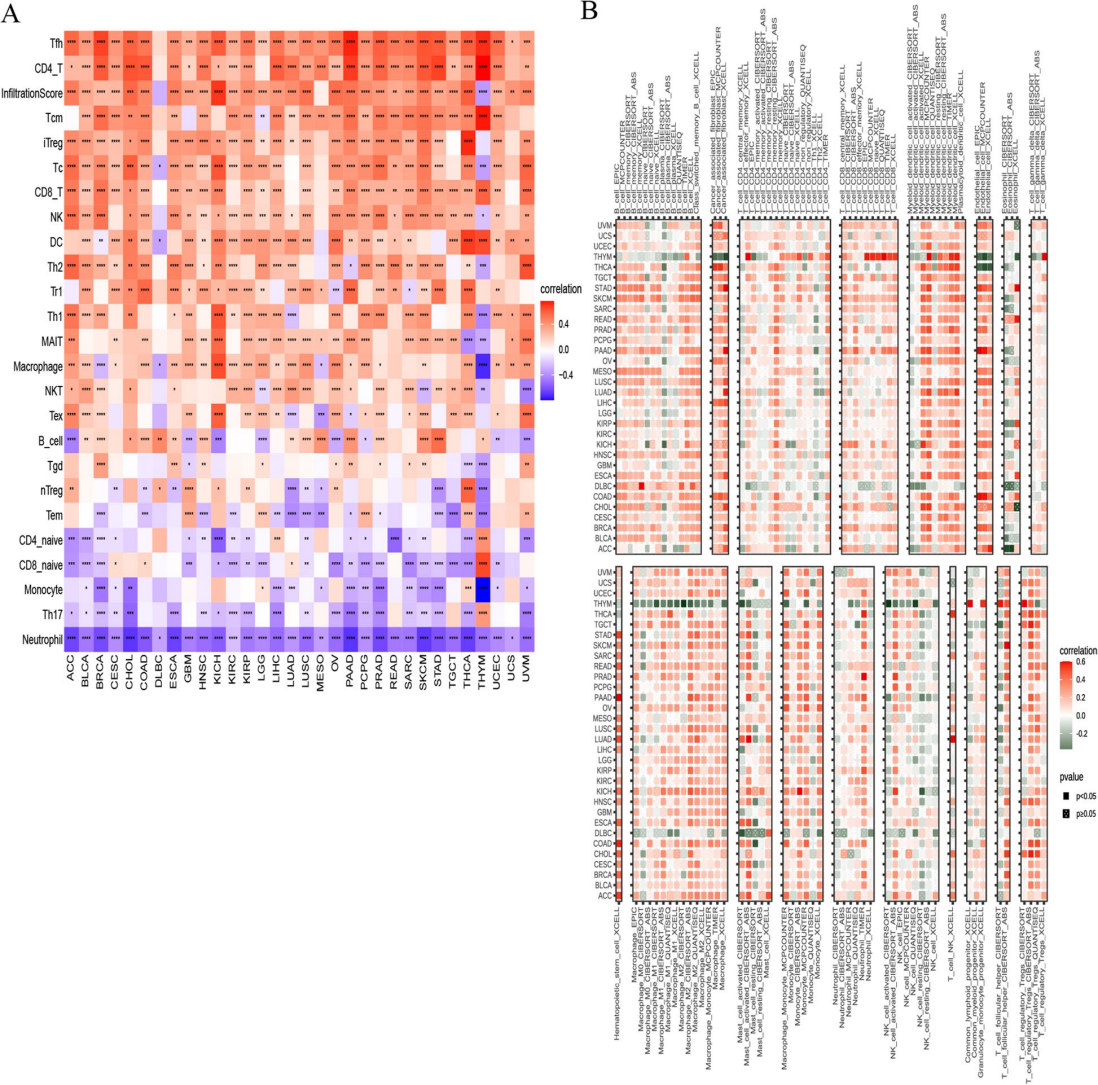
**A** Association between immune cell infiltration fractions and various malignant tumors; **B** The association between CD1C and infiltration of immune cells was evaluated using pan cancer immune infiltration data

Using data from the ImmuneCellAI and TIMER2 databases, we estimated the immune cell proportions in the TME. Difference as well as correlation analyses using the CIBERSORT algorithm were performed to complete an immune cell map of BRCA patients (Fig. 11A). The level of CD1C was positively associated with natural killer (NK) cells and T cells of infiltrating immune cells, including follicular helper T (Tfh), CD4+T, CD8+T, and regulatory T cells (Tregs). Moreover, CD1C levels were negatively correlated with naive CD4+T cells, naive CD6+T cells, and macrophage levels in BRCA. Immune cell infiltration data analysis based on various sources supported the above conclusion (Fig. 11B). These findings confirm the effects of CD1C expression on the immune activity of the TME. CD1C may increases T cell infiltration, which explains its protective role.

### Relationship of the expression of CD1C and immune-related genes

**Fig. 12:**
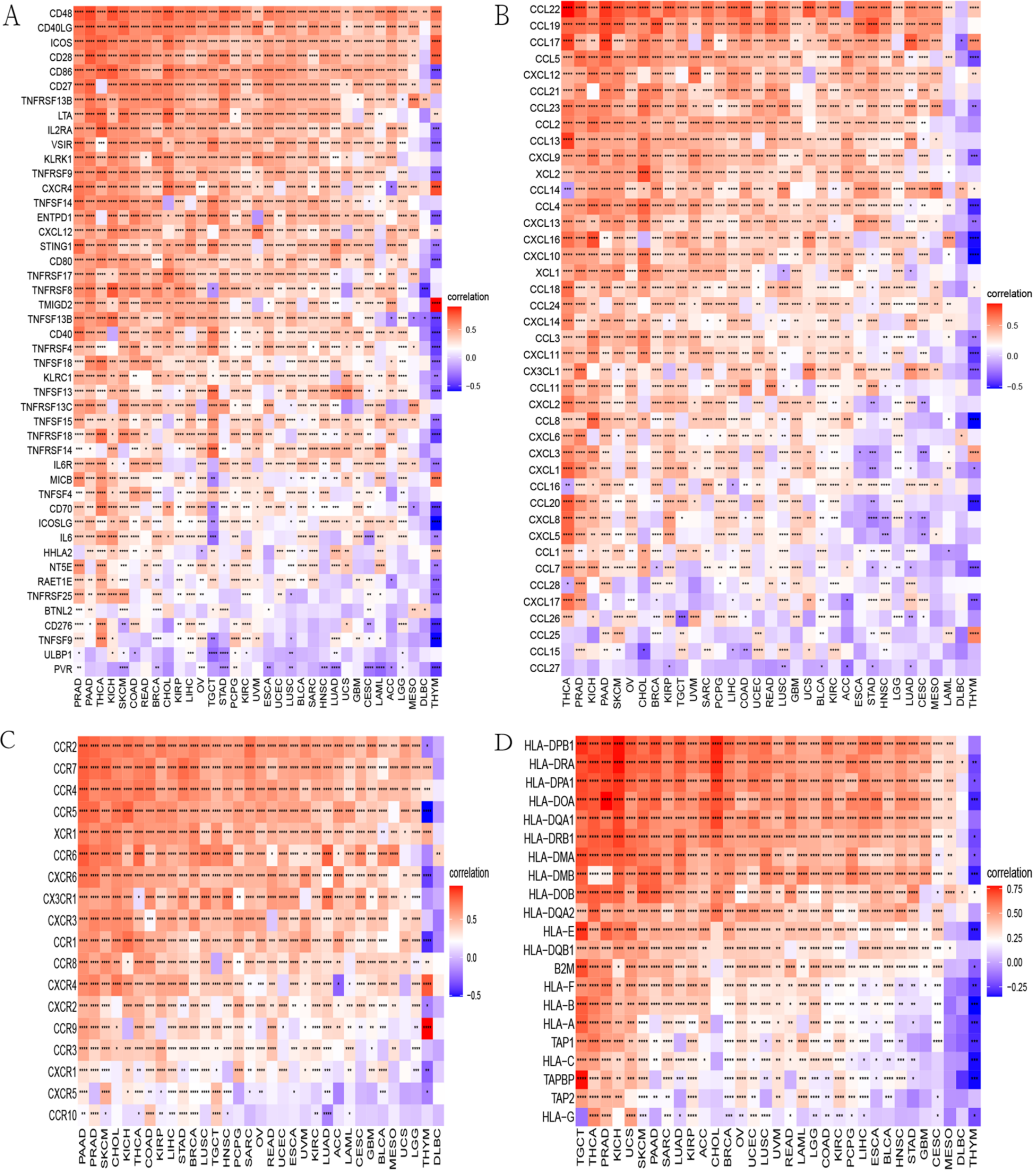
**A** Association between CD1C and immune related genes; **B** Association between CD1C and immune activating genes; **C** Correlation between CD1C and chemokines; **D** Correlation between CD1C and chemokine receptor

Gene co-expression analyses were performed and the retrieved immunologically related genes were used to further investigate CD1C-related immune functions in breast cancer. The resulting heatmap indicated that most of the immune-related genes were co-expressed with CD1C (Fig. 12A-D). This indicated that this gene is highly correlated with the immune microenvironment.

### Survival analysis of CD1C in immunotherapy patients

**Fig. 13:**
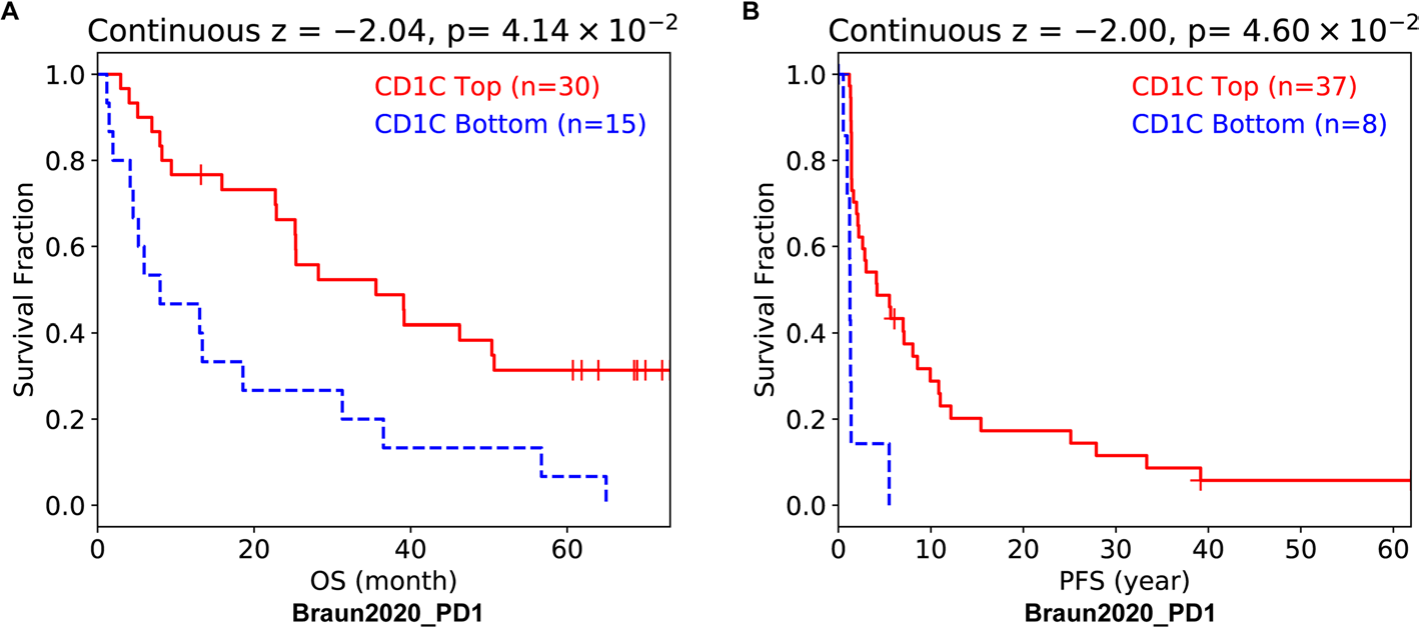
Prognosis analysis of CD1C and immunotherapy patients

Due to the lack of data on breast cancer patients, data of kidney cancer patients from the database were used to analyze the impact of CD1C on immunotherapy. The results showed that compared with patients with low expression of CD1C, patients with high expression of CD1C had better overall survival (OS) and progression free survival (PFS) after receiving PD1 treatment (Fig. 13).

### Validation of CD1C expression in BRCA

**Fig. 14:**
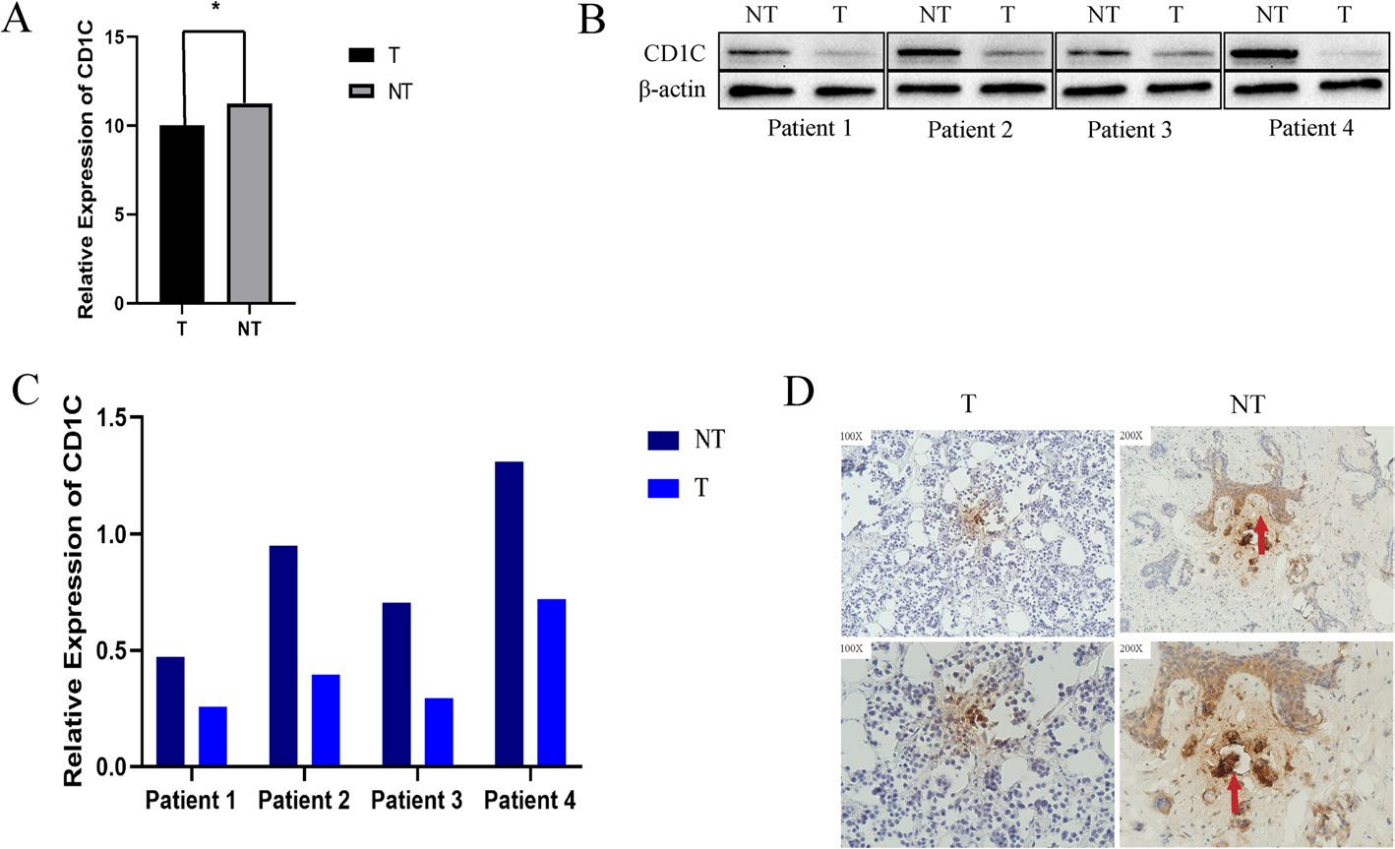
**A** mRNA expression of CD1C in tumor tissues and adjacent tissues by qRT-PCR in BRCA; **B** Western blot images of CD1C protein expression in tumor tissue and adjacent tissue in BRCA; **C** Correlation map of CD1C protein expression in tumor tissue and adjacent tissue by western blot in BRCA; **D** Representative IHC images of tumor tissues and adjacent tissues in BRCA.

PCR detection was performed on tissue samples from BRCA patients. Significant statistical differences were found in the mRNA expression of CD1C in tumor tissues and adjacent tissues (Fig. 14A). We further verified the protein expression of CD1C in BRCA samples. Western blotting results showed that the expression of CD1C protein in tumor tissues was significantly lower than that in adjacent tissues (Fig. 7C, 14B). The IHC results also confirmed this. Compared with the normal tissues near the tumor, the IHC staining of BRCA tumor tissues was weak (Fig. 14D).

### Validating the relationship between CD1C expression and clinicopathological features and prognosis in BRCA in our cohort

In the 105 BRCA patients from The Second Affiliated Hospital of Anhui Medical University, we compared differential expressions of CD1C in different subgroups based on grade, tumor stage, and molecular typing by IHC (Table 1). The present statistical analysis revealed that CD1C expression in BRCA was negatively associated with grade, tumor stage, and T classification. The difference between CD1C levels and lymph node metastasis of breast cancer was also statistically significant.

**Table 1:** Correlation between CD1C expression and clinical characteristics of BRCA patients in local hospital (*n* = 105)

Clinicopathological Characteristics	Number	High Expression	Low Expression	*P*-value
**Age (years)**				0.472
≤55	63	36	27	
>55	42	21	21	
**Grade**				0.045
1	22	15	7	
2	59	34	25	
3	24	8	16	
**Stage**				0.038
I	32	23	9	
II	59	29	30	
III	14	5	9	
**T classification**				0.021
T1	40	28	12	
T2	60	28	32	
T3	5	1	4	
**N classification**				0.233
N0	74	44	30	
N1	20	9	11	
N2	11	4	7	
N3	0	0	0	
**M classification**				
M0	57	48		
**Subtype**				0.092
Luminal A	20	13	7	
Luminal B	62	31	31	
HER-2	13	10	3	
basal	10	3	7	
**ER**				0.47
positive	80	45	35	
negative	25	12	13	
**PR**				0.793
positive	80	44	36	
negative	25	13	12	
**HER2**				0.631
positive	24	12	12	
negative	81	45	36	
**Ki-67**				0.425
≤14	28	17	11	
>15	77	40	37	
**Lymph Node Metastasis**				0.02
positive	26	9	17	
negative	79	48	31	

Among the clinical samples we collected, the onset time of breast cancer was less than five years. Under the current treatment conditions in our hospital, only a few patients relapsed or died, and there was no statistical difference after comparison. The survival curve based on clinical samples is presented in Supplementary Fig. 2.

### Validating the associations of CD1C+cells with CD4+T cells, CD4+8 cells, and CCL19

**Fig. 15:**
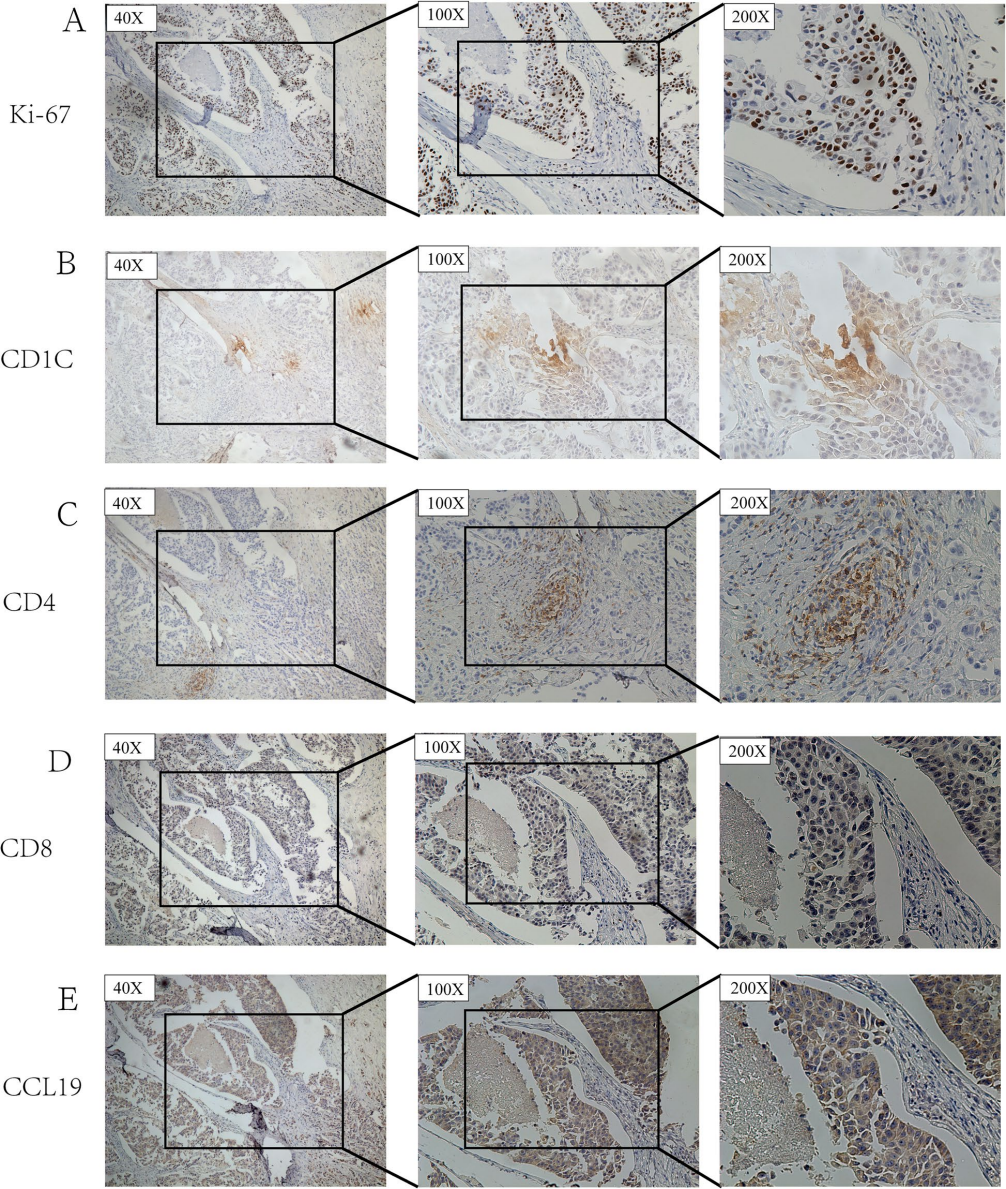
Verification of the relationship between CD1C expression and CCL19, CD4 + T cells, and CD4 + 8 cells in BRCA patients by IHC staining

Further analysis was conducted to validate the immunomodulatory effect of CD1C+cell infiltration. We performed immunohistochemical staining of CD4, CD8, CCL19, Ki-67, and CD1C in serial sections of the same tumor tissue of BRCA patients (Fig. 15). CD1C expression was negatively associated with the proliferation marker (Ki-67 expression). The level of CCL19 was markedly high in the BRCA group with high CD1C expression. Moreover, positive correlations were detected between CD1C expression and infiltration levels of CD4+ T and CD8+ T cells, indicating a key role of CD1C in regulating tumor immunology.

## Discussion

In the past decades, more and more biomarkers have been found for the diagnosis and treatment of cancer [20, 21]. However, the key regulatory factors in the BRCA microenvironment and changes in the TME in BRCA progression are yet to be clarified. In this study, public databases and new algorithms were used for bioinformatics analysis. Immune cells and stromal cells in the TME were markedly correlated with clinicopathological parameters and prognostic survival of BRCA.

Increasing evidence shows that malignant tumors are composed of tumor cells, and that surrounding non-tumor cells, known as stroma cells, change significantly [22, 23]. The TME consists of proliferating tumor cells and stroma, including TILs, fibroblasts, endothelial cells, cytokines, growth factors, and extracellular stroma. The TME changes with tumor progression and also differs in various cancer types. The composition of the TME is very important for tumor occurrence, tumor metastasis, hypoxia, and drug response [24]. Tumor cells and stromal cells mediate cancer cell growth, progression, metastasis, and drug resistance through paracrine, chemokine, and cell-cell interaction. The TME is closely associated with the complexity of tumors in breast cancer. It has been reported that IL-6 can promote the progression of breast cancer and transfer [25]. CXCL12 can increase the migration and proliferation of stromal cells in breast cancer by recruiting CXCR4, which is related to breast cancer cell lymph node metastasis [26]. Breast cancer cells secrete RANKL, which enhances breast cancer bone metastasis by increasing osteoclast activation and bone resorption [27].

The immune score was significantly correlated with clinicopathological parameters and the prognostic survival rate of BRCA, suggesting that the TME may play a key role in BRCA progression. To further investigate the mechanism of BRCA, we conducted enrichment analysis to determine the DEGs related to the TME. By screening the gene crossover between Cox regression analysis and the PPI network, we determined that CD1C was a hub gene.

CD1C belongs to the CD1 gene family, which is located outside the MHC region on chromosome 1 in humans [28]. CD1C protein is a member of MHC class I-like proteins and distributed on surfaces of DC cells [29]. CD1C plays a major role in inflammatory diseases. According to previous studies, Langerhans cell histiocytosis (LCH), COVID-19, and systemic lupus erythematosus (SLE) are related to the level of CD1C+ DC cells [10, 30, 31]. CD1C+ myeloid dendritic cells (MDC) are probable precursors of LCH disease cells, which can migrate to the disease site and differentiate into pathogenic DCs [10]. The number of CD1C+ DCs decreased significantly in SLE patients, particularly in patients with lupus nephritis [7]. CD1C has also been shown to be associated with tumors. In NSCLC, CD1C+ DCs subsets may play vital roles in anti-tumor immunity [12]. A previous study has shown that the counts of CD1C+ DCs in the blood of gastric cancer patients were increased [11]. CD1C+ DCs were found to initiate tumor specific immune reactions when cultured in vitro and injected into patients [32].

We established that CD1C expression was only slightly elevated in BRCA tissues. This may be because CD1C is mainly distributed on the surface of DC cells, which mainly exist in human blood rather than solid tissue [8]. Survival analysis indicated that high CD1C expression was related to a good prognosis in BRCA patients. Biological annotation of the GO database showed that our target genes were mainly related to immunity, including cell killing and cytokine production. KEGG database analysis indicated that CD1C is related to the immune-related pathway. Reactome is also an immune-related database, which showed that CD1C is particularly involved in immunoregulatory interactions between non-lymphoid and lymphoid cell pathways.

A high proportion of CD4+T and CD8+T cells is a vital factor leading to better OS in the high CD1C group. According to previous studies, CD1C+ DCs can present antigens to CD4+T and CD8+T cells [8]. Our immunohistochemical test verified that CD1C levels were positively correlated with CD4+T and CD8+T cells. CD8+T cells, NK cells, and CD4+T cells are important participants in the anti-tumor process [33]. CD1C levels were positively correlated with CD8+T, CD4+T, and NK cells, explaining the protective roles of CD1C in breast cancer. T cells help malignant tumor cells evade attack from cytotoxic CD8+T cells [34]. We determined that T infiltration levels were positively correlated with CD1C, implying that the function of CD8+T cells may be limited. DCs are involved in CD8+T cell priming [35]. CXCL9 produced by DCs and CXCL10 interacted [36]. DCs express CD86ligands for binding to their respective CD27 and CD28 receptors on CD8+T cells. These interactions were used to activate and prime CD8+T cells [37]. CXCL9, CXCL10, CD86, CD27, and CD28, which appeared in the above processes, were all positively associated with CD1C expression. This implies that CD1C may participate in CD8+T cell-mediated anti-tumor immunity.

CCL19 can home T cells to secondary lymphoid organs [38]. Elevated CCL19 levels in a tumor result in anti-cancer TIL infiltration [39]. Our results confirmed that elevated CCL19 levels positively correlated with high CD4+T and CD8+T cell infiltration in the TME of BRCA. Our experiments also verified the positive association between CD1C and CCL19 expression. This indicated that CCL19 may be involved in the antitumor mechanism of CD1C, but the specific mechanism is not clear.

This study has several limitations. First, there is no detailed treatment information for breast cancer patients in the TCGA database. Therapeutic efficacy is a potential confounding factor. Secondly, survival rates for breast cancer patients are usually very high. The five-year survival rate of breast cancer patients treated in our hospital can reach more than 80%. Therefore, clinical trials need a long follow-up period to further judge the impact of CD1C on prognosis and survival. Finally, although the effect of CD1C on immunotherapy in patients with renal cell carcinoma was confirmed by the TIDE database, the impact of CD1C on immunotherapy is not clear in breast cancer patients. In the next study, this problem will be studied by collecting more clinical samples.

## Conclusion

Based on immune and stromal scores, we conclude that the TME plays an important role in BRCA initiation and progression. Moreover, CD1C was identified as a hub gene. In summary, CD1C is a prognostic biomarker for breast cancer and a potential treatment target.

## Supplementary Information

**Additional file 1: Figure 1.** PPI network and univariate Cox regression analysis. **Figure 2.** Survival analysis curve of TLR7, Mrc1, CCL19, cd3e, CD1C, CD1e, IL2 in BRCA. **Figure 3.** Relationship between TLR7, Mrc1, CCL19, cd3e, CD1C, CD1e, IL2 and clinical characteristics of BRCA. **Table 1. **The primer sequences of CD1C.

## Data Availability

Publicly available datasets were analyzed in this study. All data can be found as specified below: RNA sequencing data was downloaded from the TCGA database (https://www.cancer.gov/) through the UCSC XENA website (https://xena.ucsc.edu/). The GSE20698 dataset was downloaded from the Gene Expression Omnibus (GEO) database (https://www.ncbi.nlm.nih.gov/geo/). The data of protein-protein interactions were derived from the String database (https://string-db.org/). The tumor immune infiltrating cell data were obtained from the ImmueCellAI (http://bioinfo.life.hust.edu.cn/ImmuCellAI#!/) and TIMER2 databases (http://timer.comp-genomics.org/). Data of patients receiving immunotherapy were downloaded from the TIDE database (http://tide.dfci.harvard.edu/). All datasets are open-access datasets.
