# Supplementary material for: CD1C is associated with breast cancer prognosis and immune infiltrates
Source: BMC Cancer. 2023 Feb 8;23:129. doi: 10.1186/s12885-023-10558-2 (PMC9905770; doi:10.1186/s12885-023-10558-2)
Supplement: Supplementary file 1 — Additional file 1: Figure 1. PPI network and univariate Cox regression analysis. Figure 2. Survival analysis curve of TLR7, Mrc1, CCL19, cd3e, CD1C, CD1e, IL2 in BRCA. Figure 3. Relationship between TLR7, Mrc1, CCL19, cd3e, CD1C, CD1e, IL2 and clinical characteristics of BRCA. Table 1. The primer sequences of CD1C. [file 12885_2023_10558_MOESM1_ESM.pdf]

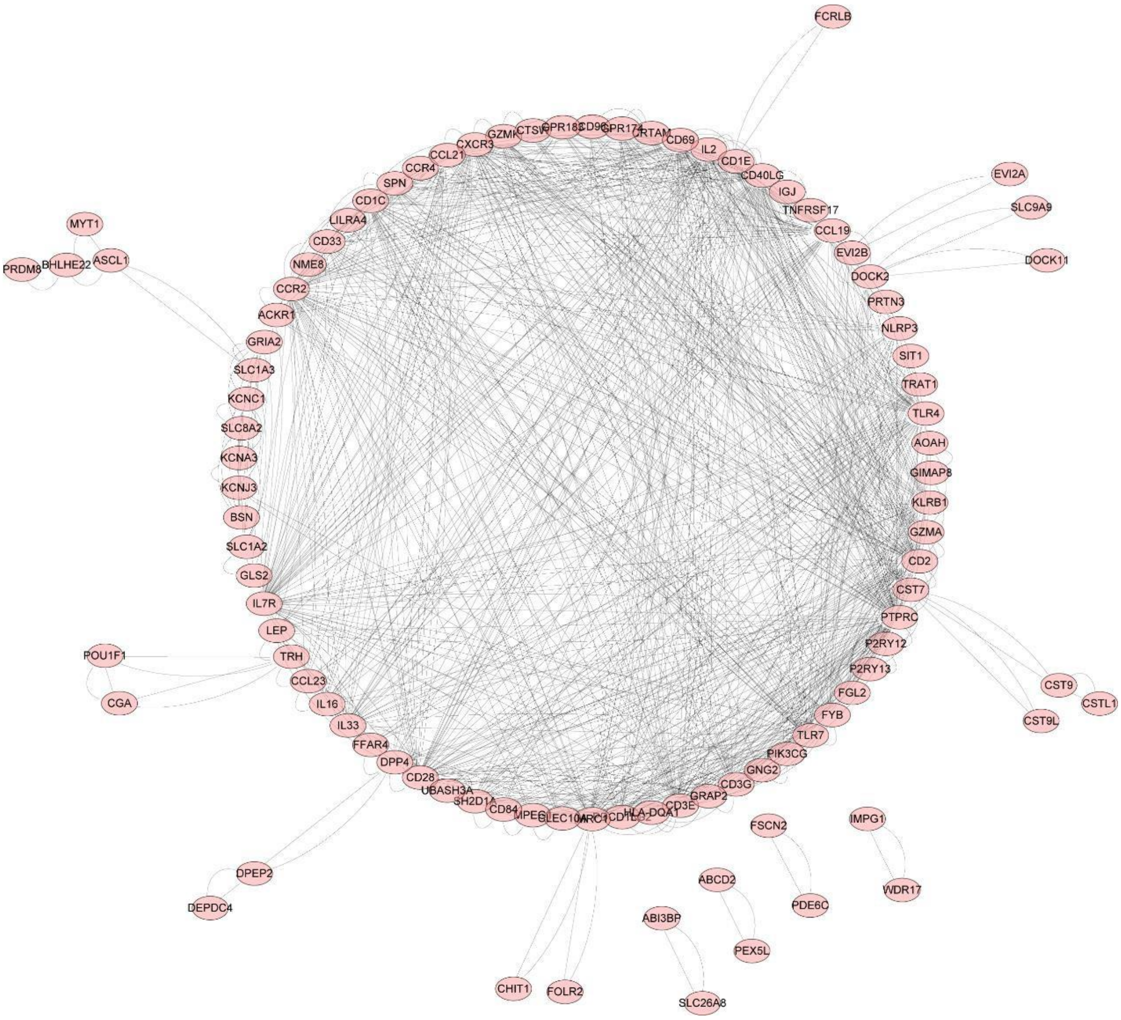

Supplementary Figure 1: PPI network and univariate Cox regression analysis.

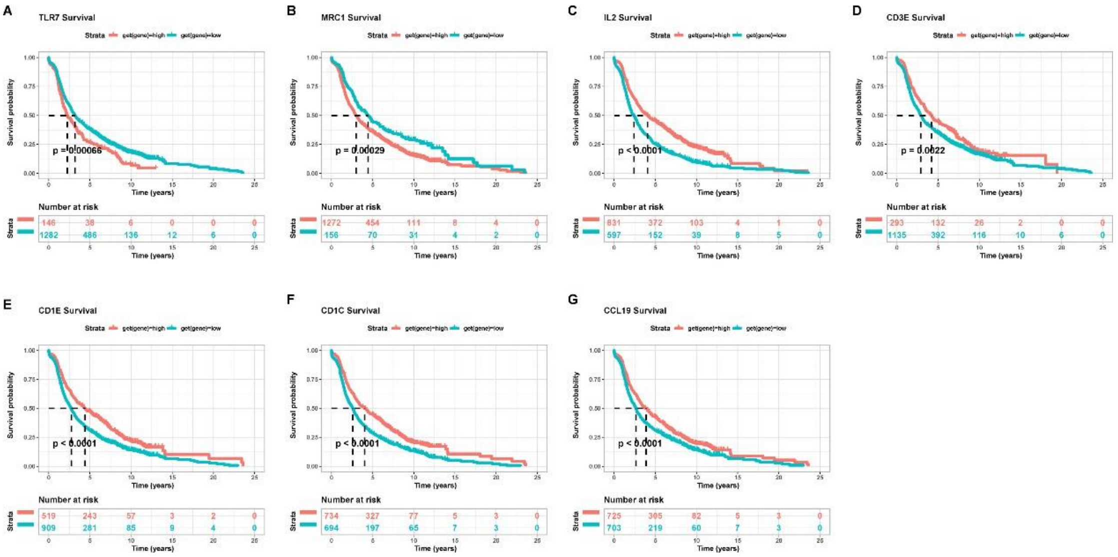

Supplementary Figure 2: Survival analysis curve of TLR7, Mrc1, CCL19, cd3e, CD1C, CD1e, IL2 in BRCA.

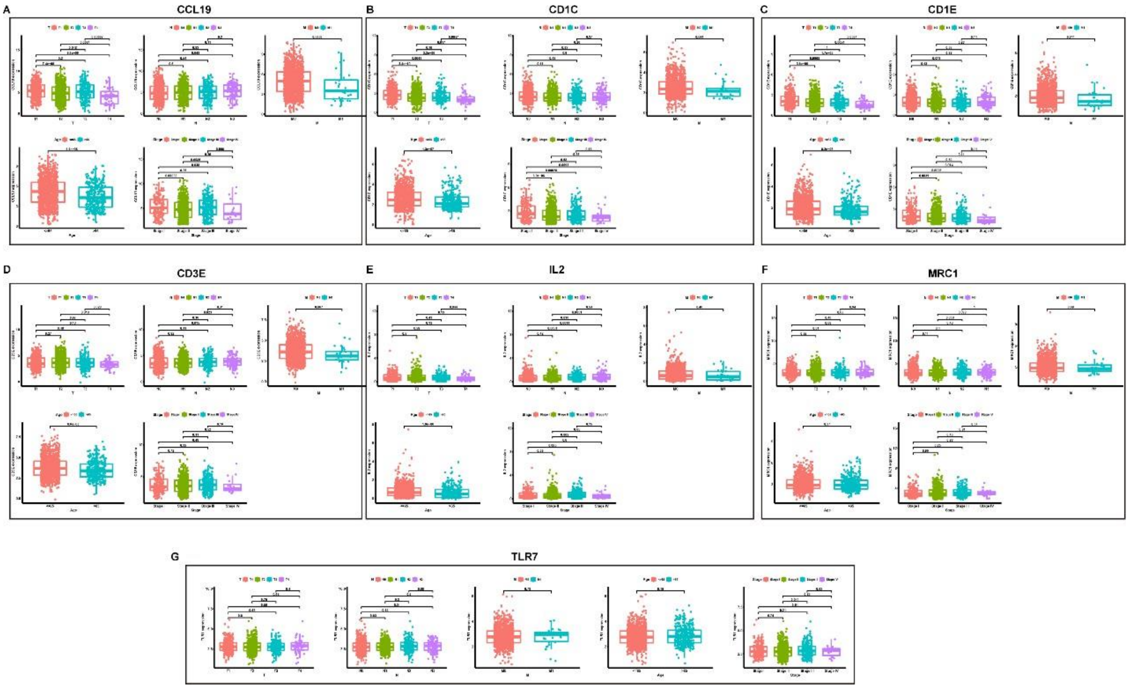

Supplementary Figure 3: Relationship between TLR7, Mrc1, CCL19, cd3e, CD1C, CD1e, IL2 and clinical characteristics of BRCA.

Table 1：The primer sequences of CD1C

Name	Forward primer (5′-3′)	Reverse primer (5′-3′)
CD1C	GGAGAGAGCGTTCCAGACAGC	GGTGCTCCACTTCCCTTCATC
